# Identification of Gingival Crevicular Fluid Sampling, Analytical Methods, and Oral Biomarkers for the Diagnosis and Monitoring of Periodontal Diseases: A Systematic Review

**DOI:** 10.1155/2016/1804727

**Published:** 2016-12-15

**Authors:** Zeyad Nazar Majeed, Koshy Philip, A. M. Alabsi, Saravanan Pushparajan, Dasan Swaminathan

**Affiliations:** ^1^Department of Restorative Dentistry, Faculty of Dentistry, University of Malaya, Kuala Lumpur, Malaysia; ^2^Department of Periodontology, Faculty of Dentistry, University of Babylon, Babylon, Iraq; ^3^Institute of Biological Sciences, Faculty of Science, University of Malaya, Kuala Lumpur, Malaysia; ^4^Department of Oral and Craniofacial Sciences, Faculty of Dentistry, University of Malaya, Kuala Lumpur, Malaysia

## Abstract

*Background*. Several studies in the last decades have focused on finding a precise method for the diagnosis of periodontal disease in its early stages.* Aim*. To evaluate from current scientific literature the most common and precise method for gingival crevicular fluid (GCF) sample collection, biomarker analytical methods, and the variability of biomarker quantification, even when using the same analytical technique.* Methodology*. An electronic search was conducted on in vivo studies that presented clinical data on techniques used for GCF collection and biomarker analysis.* Results*. The results showed that 71.1%, 24.7%, and 4.1% of the studies used absorption, microcapillary, and washing techniques, respectively, in their gingival crevicular fluid collection. 73.1% of the researchers analyzed their samples by using enzyme-linked immunosorbent assay (ELISA). 22.6%, 19.5%, and 18.5% of the researchers included interleukin-1 beta (IL-1*β*), matrix metalloproteinase-8 (MMP-8), and tumor necrosis factor-alpha (TNF-*α*), respectively, in their studies as biomarkers for periodontal disease.* Conclusion*. IL-1*β* can be considered among the most common biomarkers that give precise results and can be used as an indicator of periodontal disease progression. Furthermore, paper strips are the most convenient and accurate method for gingival crevicular fluid collection, while enzyme-linked immunosorbent assay can be considered the most conventional method for the diagnosis of biofluids.

## 1. Introduction

The aim of this review is to evaluate GCF sampling, analytical methods, and oral biomarkers used for the diagnosis and monitoring of different periodontal diseases. It also attempts to explore the main reasons for differences in biomarker quantification among the investigators, by reviewing studies based on inclusion and exclusion criteria from January 2005 to May 2015.

Periodontal diseases are multifactorial infections influenced by the interaction of different types of bacteria with host cells and tissues leading to the release of many cytokines and chemokines which cause the destruction of the periodontal structures [1]. GCF in subjects with periodontal disease contains inflammatory cells, bacteria, tissue breakdown products, antibodies, and complement system proteins and enzymes, in addition to many inflammatory mediators [2]. GCF can be considered among the most nontraumatic investigational methods used to provide information about periodontal tissue conditions, including the status of the connective tissue and the degree of hard tissue destruction [3]. The severity of periodontal tissue inflammation can be estimated by measuring proinflammatory indicators such as IL-1, -6, and -8 and TNF-*α* that have an important role in the pathogenesis of periodontal diseases [4].

Periodontal diseases must be described by a criteria that is clear and suitable for any examiner to apply, so that the same diagnosis can be reached by other examiners under same conditions [5]. The early diagnosis of periodontal diseases is of significant importance that demands a quick, sensitive, and precise chair-side analytical test [6, 7].

The methods of diagnosis should provide relevant information to assist the differentiation among a variety of periodontal diseases, the degree of periodontal tissue destruction, and the prognosis of periodontal disease. Nowadays, the basic method used in the diagnosis of periodontal diseases depends mainly on the measurement of the clinical periodontal parameters including plaque score (PS) or plaque index (PI), clinical attachment loss (CAL), probing depth (PD), gingival index (GI), or bleeding on probing (BOP), in addition to radio graphical findings. However, the findings from these clinical parameters provide evidence regarding the previous damage of the periodontium rather than clarifying the future condition of the periodontal tissue. Therefore, it is very important to find a method that can predict upcoming periodontal disease. More reliable techniques for periodontal diagnosis need to be researched [105] such as the MMP-8 chair-side testing that depends on the immunochromatography principle which could be beneficial in supporting the clinical diagnostic parameters during the maintenance phase [6, 77]. Moreover, the results from such a test increase the accuracy of the periodontal disease diagnosis and results of unsuccessful treatments [106]. However, this test is fast, inexpensive, and easily performed and takes just 5 minutes [107].

## 2. Methodology

### 2.1. Inclusion and Exclusion Criteria

Inclusion criteria in the current study included (a) clinical studies, (b) studies performed on both systemically healthy and systemically unhealthy subjects [patients with diabetes mellitus (DM), coronary heart disease, or rheumatoid arthritis (RA)] with periodontal disease, (c) studies performed on smokers, anxious, or pregnant subjects, (d) studies published only in the English language, and (e) studies clarifying the methodology for GCF sample collection and biomarkers analytical techniques. Exclusion criteria included studies specifically designed to investigate the biomarkers in peri-implant sulcus fluid (PISF), letters to the editor, historic reviews, and commentaries.

### 2.2. Search Protocol

**Figure 1:**
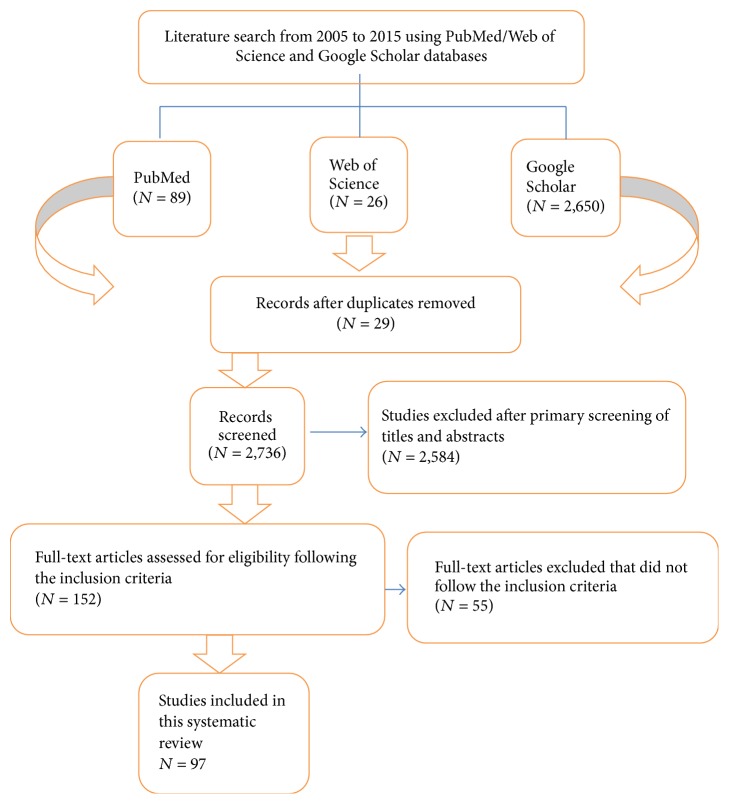
Flow chart for research strategy.

PubMed, Google Scholar, and Web of Science databases from 2005 to 2015 were searched using combinations of the following keywords: “periodontal disease”, “periodontitis”, “oral biomarkers”, “biomarkers”, and “gingival crevicular fluids”. Titles and abstracts of studies were identified using the above-described protocol, selected by the authors, and checked for agreement. Full texts of the studies were determined by title and abstract and then independently assessed by the authors (Zeyad Nazar Majeed, Dasan Swaminathan, A. M. Alabsi, Koshy Philip, and Saravanan Pushparajan) with reference to the inclusion and exclusion criteria. Initially 2,765 publications were identified, while only 97 studies which fulfilled the inclusion criteria were included and processed for data extraction as shown in the flow chart (Figure 1).

## 3. Gingival Crevicular Fluid Collection Methods

### 3.1. Intracrevicular Washing Technique

The device used to perform this method consists of two injection needles placed one inside the other. This method for GCF collection was well described by Salonen and Paunio [108] and the main details of the studies that utilized washing technique in their GCF collection were briefly illustrated in Table 1.

### 3.2. Microcapillary Technique

A calibrated volumetric or noncalibrated microcapillary pipette with known volume is used to collect GCF. Pradeep et al. [23, 24] gave a good description in their studies on how to perform this collection technique. In this systematic review, 24 studies used this technique (Table 2).

### 3.3. Absorption Technique

**Figure 2:**
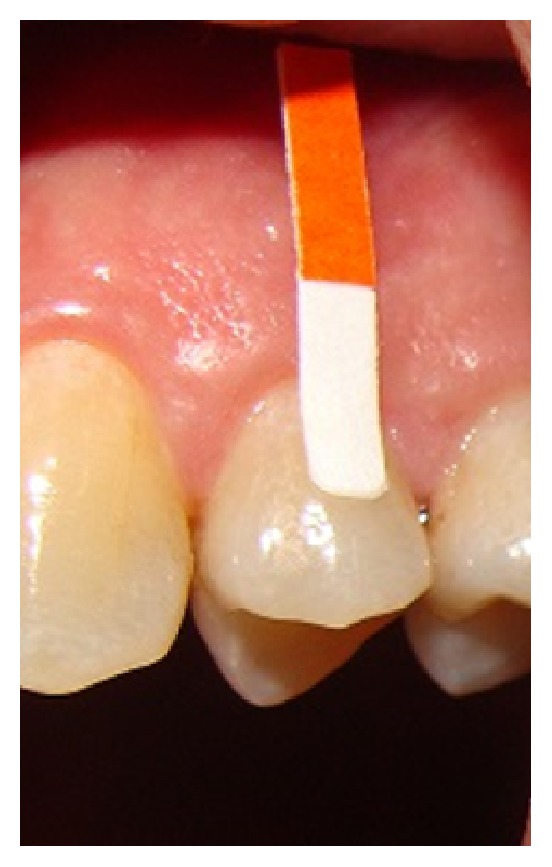
Extracrevicular GCF collection.

**Figure 3:**
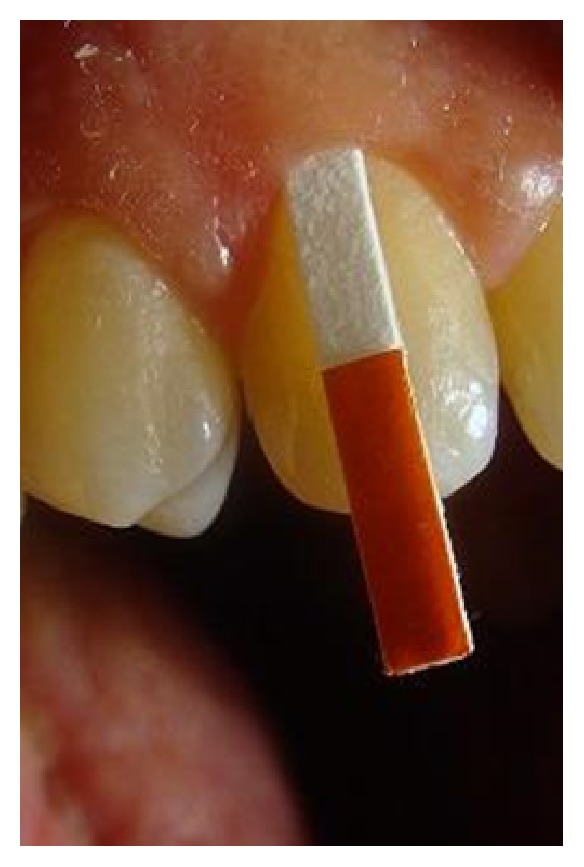
Intracrevicular GCF collection.

Table 3 summarized the most important findings of the studies that used the absorption technique. Generally, this technique is divided into extracrevicular (Figure 2) and intracrevicular (Figure 3). The first one is performed by placing paper strips over the gingival crevice to reduce trauma. The second method is the intracrevicular technique which is the most commonly used. It may be subdivided into superficial and deep, depending on the depth of strip insertions into gingival sulcus or periodontal pocket [109].

## 4. Results

A meta-analysis of the results was not carried out in this systematic review because the heterogeneity of the reviewed studies involved the following aspects: GCF sampling, analytical methods, and oral biomarkers used in the diagnosis and monitoring of periodontal disease.

### 4.1. Sampling Methods

With regard to the techniques used for GCF collection, 97 studies were included and illustrated in our review (Tables 1, 2, and 3). 69 studies used paper strips for GCF collection (Table 3), 24 studies used microcapillary pipettes (Table 2), and only 4 studies utilized the gingival washing method to collect their samples (Table 1).

### 4.2. Analytical Methods

As shown in Tables 1, 2, and 3 many methods were employed to analyze GCF samples in order to get the desired results. The ELISA analytical method was clearly the preferred method in the majority of the studies. Seventy-one out of the 97 studies that we reviewed used the ELISA technique to analyze the GCF samples.

### 4.3. Biomarkers

Of the 97 studies, IL-1*β* (22 studies), MMP-8 (19 studies), and TNF-*α* (18 studies) were reviewed. These results indicated that these biomarkers concentrations could be used to compare the different stages of periodontal disease and/or assess the effectiveness of periodontal therapy.

**Figure 4:**
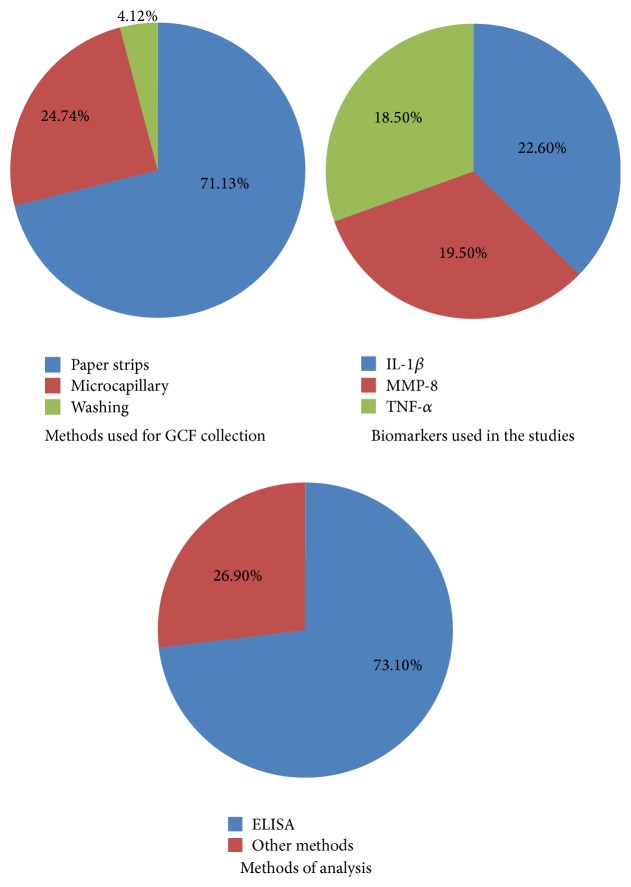
Results summary.

Results on sampling and analytical methods and biomarkers are summarized in Figure 4.

### 4.4. IL-1*β*

A comparison of GCF IL-1*β* levels between periodontally diseased patients and healthy subjects was conducted in order to explain the suitability of using IL-1*β* as a biomarker for periodontal disease progression. In this comparison, only 10 studies among the 22 studies were included (Table 4), due to the exclusion of the studies that did not show enough clinical data and the studies that were conducted on periodontally diseased patients with systemic diseases (e.g., diabetes mellitus) or any other conditions that might affect the results (e.g., pregnant women and smokers). IL-1*β* concentrations in all studies showed moderate to high significant differences between the healthy subjects and patients with CP or GAgP.

Of the 10 studies that were included to determine the difference in IL-1*β* levels, only 3 studies [41, 51, 103] showed differences in IL-1*β* levels between healthy subjects and patients with gingivitis. However this difference was nonsignificant.

### 4.5. MMP-8

For MMP-8 levels differences between healthy and diseased subjects, only 4 studies fulfilled the inclusion criteria, the same as for IL-1*β*. All the studies (Table 5) showed differences between healthy subjects and CP patients. Although these differences were highly significant in the studies done by Konopka et al. [67], Rai et al. [88], and Teles et al. [98], it was not considered significant by Yakob et al. [11].

### 4.6. TNF-*α*

For TNF-*α* levels differences between healthy and diseased subjects, only 8 studies were included which fulfilled the inclusion criteria (Table 6). Reis et al. [91] showed that TNF-*α* levels were significantly higher in diseased sites compared to nondiseased sites. Gokul [14] showed a highly significant difference between healthy subjects and patients with gingivitis and CP. Kurtiş et al. [70] showed highly significant difference between healthy subjects and patients with CP and GAgP. However, the other studies showed only slight difference in TNF-*α* level between healthy and diseased subjects.

Despite the similarity in results with the majority of studies reviewed in this systematic review, there were still different effects of biomarkers on periodontal disease. The findings of the studies also determined how those biomarkers could be utilized in the diagnosis or monitoring periodontal disease status, as shown in Tables 4, 5, and 6. The present study showed that there was variability in the findings between investigators even when using the same analytical techniques. In order to clarify the differences in outcome, a comparison was made among the studies that used the same analytical methods (Tables 7 and 8).

As shown in Table 7, only five studies were included that used the same analytical method (standard ELISA technique) for quantitative determination of IL-1*β* concentrations. It was quite noticeable that there were differences in the values of the mean IL-1*β* concentrations between the studies within the same study group (H, G, CP, and GAgP). For example, the mean IL-1*β* concentration among healthy subjects in the five studies showed differences in mean values: 49.81, 195.77, 36.44, 15.5, and 17.8732.

Only two studies were involved for comparing MMP-8 mean values (Table 8). Both showed relatively close results in which there was a slight difference in the mean values of MMP-8 in each study group (H, CP).

## 5. Discussion

This systematic review was designed to discover the most common and accurate GCF collection and biomarker analytical methods and to determine the reliable biomarkers that could be used to detect periodontal disease.

### 5.1. GCF Sampling Methods

This paper discussed the three main methods of GCF collection: absorption, microcapillary pipetting, and the washing method. There were variations in GCF collection techniques in several of the clinical studies that were reviewed.

#### 5.1.1. Absorption Method

The differences could be summarized as follows:

- The majority of the studies used paper strips which were considered to be more efficient in GCF collection because they could be inserted easily into gingival sulcus or periodontal pockets, as well as for their ability to absorb fluids. However, few studies used paper points (size 30) to collect GCF samples although it was shown that paper points and paper strips had different absorption rates. A study done by Guentsch et al. [110] indicated that cytokine levels were higher when paper strips were used. Paper points are more commonly used for subgingival plaque collection in microbiological analysis.
- The time in which the paper strips or paper points were left in the sulcus varied between 30 seconds [68, 76, 111] and 1 minute [112]. The period most frequently used was 30 seconds to decrease the risk of blood or saliva contamination.
- There were variations in the sites from which the GCF samples were collected. Many studies collected GCF samples from diseased sites only [72] in patients with periodontal disease, while other researchers collected samples from both healthy and diseased sites [111].

It was thus important to note that the majority of the studies showed that biomarker levels positively correlated with the periodontal parameters (GI, PD, and CAL). At the same time, it was clarified that healthy sites in individuals with periodontal disease showed increased concentrations of biomarkers in comparison to healthy sites in subjects without periodontal disease. This could be because biomarkers were affected by the bacterial composition of the neighboring subgingival plaque [98] and the fact that the development of periodontitis was site-specific [53].

#### 5.1.2. Microcapillary Pipetting

The time needed to collect GCF samples was related to the desired amount of GCF required and also to the condition of the sample sites (diseased or healthy). The majority of clinical studies collected GCF samples by keeping the microcapillary pipettes at the entry of the pocket for 10 minutes [22, 85]. From our experience, this duration was sufficient if we collected the GCF samples from diseased sites. However, collection of the samples from healthy subjects or healthy sites in patients with periodontitis required 30–50 minutes. This difference in collection time between healthy and diseased sites was due to the flow of GCF which was positively related to the severity of periodontal disease [113–115]. The lengthy duration needed to collect GCF when using microcapillary pipettes was considered one of the limitations of this technique, which could increase the possibility of saliva and blood contamination. It would also require more effort from the clinician and could be time-consuming for the subjects.

#### 5.1.3. Washing Method

The results of this review showed that the washing technique of GCF collection were not common due to the technique sensitive difficulties. Also, there was a high rate of blood contamination due to the increased possibility of gingival irritation.

### 5.2. GCF Analytical Method

In the absence of convincing evidence and the deficiency of data from well-designed studies that focused on the techniques used in the analysis of GCF and after determining the advantages and disadvantages of each technique, it was still difficult to declare that a specific technique was better than others. This was especially so if we considered the following aspects: (a) accuracy and efficiency in biomarker detection and quantification, (b) feasibility, (c) cost, and (d) time. It was clearly shown that the majority of researchers used the ELISA technique in their clinical studies, probably due to its simplicity. It is quite important to clarify that the use of ELISA is not the most accurate technique. For example, Leppilahti et al. [73] showed that the IFMA technique is more accurate than using ELISA.

### 5.3. Oral Biomarkers for Periodontal Disease Analyzed from GCF Sampling

To date, an accurate diagnosis depends mainly on clinical periodontal examination, radiographic examination, and laboratory tests for microbial analyses [116] that permit a precise evaluation and analysis of bone and attachment loss levels. These findings could be supplemented by GCF analyses where, as many studies have suggested, GCF is a source of bimolecular sampling to investigate the condition of periodontal tissues [112, 117]. GCF ingredients are composed of many components that have been described as markers for periodontal disease development. These comprise host-derived enzymes, host-response modifiers, and tissue breakdown products [64].

It is known that biomarkers are objective and measurable characteristics of biological processes [118] and they can support clinical evaluation, that is, if we fully understand the normal physiology of the biological processes of periodontal disease diagnosis and progression [119]. There are many biomarkers that can be derived from different biofluids such as blood, serum, saliva, and GCF and from different sources such as microbial dental plaque biofilm, connective tissue breakdown products, inflammatory mediators, and host derivatives. For example, MMPs that exist in GCF, saliva, mouth-rinses, and peri-implant sulcular fluid (PISF) can be used to discover a novel chair-side and point-of-care analytical test, which is a nontraumatic method for the diagnosis of periodontal diseases [120, 121].

In this review we focused on GCF biomarkers because of their close proximity to periodontal tissue which minimizes the possibility of reflecting a response on other inflammatory processes in the body.

Several studies have suggested that IL-1*β* levels can be used as a good biomarker to differentiate between healthy and chronic periodontitis sites [12, 41, 51, 53, 98]. They can also be used to discriminate healthy subjects from patients with AgP [41, 51, 80, 99]. Owing to the slight differences in IL-1*β* levels between healthy and gingivitis sites [41, 51, 103], it is difficult to use them as indicators or predictors for disease initiation from healthy status to gingivitis.

Much greater levels of MMP-8 in GCF have been observed in periodontitis patients than in healthy subjects [6, 67, 88, 98, 122, 123]. This variation in MMP-8 levels can serve as an indicator for periodontal disease development. Furthermore, Leppilahti et al. [72] found that the levels of MMP-8 in GCF at baseline can predict the behavior of MMP-8 levels during the phase of maintenance.

Yakob et al. [11] however found that there were no statistical differences between healthy and diseased groups, attributing these findings to the differences in the methods used for GCF collection.

The results of this review, as also indicated in many studies, showed minimal increase in TNF-*α* levels from healthy to periodontally diseased sites [59, 80, 99, 103]. In other studies there was substantial elevation in TNF-*α* concentration from healthy to diseased sites [14, 70, 91]. Thus, TNF-*α* concentrations may also be used as a predictor of disease progression.

This systematic review aimed to explore the most reasonable factors that lead to variability in the findings among different studies even when using the same analytical techniques. In order to achieve this, a comparison between the mean values of two biomarkers (IL-1*β* and MMP-8) was conducted (Tables 7 and 8). The studies that comprised sufficient data such as sample numbers, clear analytical techniques, number or amount of GCF samples, and accurate assessment of the clinical diagnosis through the use of clinical diagnostic parameters (PD, CAL, PI, BOP, and GI) were included in this comparison. Studies that included smokers and diabetic subjects were excluded to minimize the effects on the results. For instance, clinical studies in different populations showed that smoking increased the risk of periodontitis and also that smokers had higher progression and severity of periodontal disease [124]. Tymkiw et al. [102] found that smoking inhibited the expression of many biomarkers including proinflammatory chemokines, regulators of T-cells, and natural killer cells. This inhibition resulted in a decrease in the recruitment of many proinflammatory cytokines and cells to the periodontally inflamed sites, which caused unsuccessful protection against bacterial invasion.

Furthermore, the mechanisms that explain the association between diabetes and periodontitis are not fully understood but encompass aspects of immune function, inflammation, cytokine biology, and neutrophil activity [125]. Types 1 and 2 diabetes have been related to elevated levels of inflammatory mediators [126], such as IL-1*β* [127] and TNF- *α* [128].

Table 7 shows a wide variety in the mean values of IL-1*β* concentrations in the studies. However, the majority of investigators used similar parameters such as size of study population which may affect mean and standard deviation, GCF collection methods (mainly paper strips), analytical techniques (standard ELISA), and clinical diagnostic parameters to categorize the study sample (mainly PD, CAL, PI, and BOP). However, we have noticed that companies manufacturing ELISA kits apply different protocols for measurements of biomarkers and the kit reagents may vary in their detection ability. Another contributing factor to this variability may be the difference in the amount of the collected GCF fluids, ranging from 1–4 paper strips collected from each subject. Such differences in GCF volumes may also cause wide variation in detection rates of biomarkers. This can be supported by the results in Table 8 which showed slight differences between the biomarker mean values, as both studies used the same ELISA kit protocol (R & D System) and an equal number of GCF samples (1 paper strip).

## 6. Limitations

Due to the heterogeneity of strategies used in the reviewed studies such as sampling, analytical methods, and biomarkers used, a meta-analysis of the results was not possible.

## 7. Conclusion

In the case of GCF collection methods, paper strips are the easiest and a more precise method. For GCF sample analysis, it is difficult to determine the most accurate method of analysis, but this review has noted that the majority of researchers depended on ELISA technique.

It can be concluded that it is better to use more than one biomarker in determining the inflammatory activity of periodontal disease. IL-1*β* and MMP-8 can be considered the most preferred cytokines for determining inflammatory activity in the periodontium.

The collected GCF volume and different ELISA kit manufacturing companies are the major causative factors for variation among the investigators.

In general, it is still early to depend on oral biomarkers alone in the diagnosis of periodontal disease, especially in the absence of universal methods for the collection and analysis of these biomarkers. However, it can be utilized to support the clinical parameters which are the most reliable diagnostic methods and also for monitoring periodontal disease progression.

## Recommendations for Future Studies

The aim for investigating oral biomarkers is to discover the possibility of using them in the prediction, diagnosis, and monitoring of periodontal diseases or at least to be used as adjunctive to traditional periodontal examination and diagnosis. We believe that, in order to achieve this, researchers should take into consideration the following recommendations in their future studies:

1. The measurement of GCF biomarkers levels should be done by using different collection and analytical methods in order to determine the most accurate technique that can be standardized universally.
2. Comparison of GCF biomarkers levels in different ethnic groups consisting of large sample size should be considered in order to explore the effects of genetic differences on biomarkers levels and also to enable proper statistical analysis.
3. The choice of biomarkers in GCF derived studies is important. Biomarkers that are known to influence the prediction and progression of periodontal diseases should be investigated for statistical correlation to each other. Examples of biomarkers which can contribute to this are IL-1*β* and MMP-8 whereby Salminen et al. [129] used three salivary biomarkers together to diagnose periodontal disease.

## Figures and Tables

**Table 1:** Summary of washing techniques used in the GCF collection.

Reference	Analysis	Markers	Aim	Main findings
[8]	ELISA, spectrophotometer (SPM)	IL-1*β*, IL-18, elastase activity	To evaluate inflammatory activity in GCF in RA patients and patients without RA. To determine if the periodontal inflammation reduced after RA treatment.	RA anti-inflammatory treatment reduced periodontal inflammation.

[9]	Capillary zone electrophoresis coupled with laser induced fluorescence detection (CZE-LIFD)	Arginine, glutamate	To evaluate the glutamate and arginine GCF levels in adult chronic periodontitis (CP) patients against healthy controls. To compare two types of microdialysis probes: U-shaped probes and normal.	Arginine level was elevated and glutamate level was decreased in CP patients, compared to healthy subjects. No statistical differences were found between the U-shaped and normal probes.

[10]	Polymerase chain reaction	Host *β*-globin gene fragments	To determine the expression of gene fragments of the host *β*-globin in GCF at different stages of periodontal disease.	Periodontal diseases have marked effect on gene fragment expression in GCF. Thus *β*-globin DNA could be used as a biomarker for periodontal disease.

[11]	ELISA	MMP-8, MMP-9	To determine the association between the existence of subgingival microorganisms in certain locations and the GCF levels of MMP-9 and MMP-8.	The existence of subgingival microorganisms in GCF, mainly *T. denticola, *increased the MMP-9 and MMP-8 levels.

**Table 2:** Summary of microcapillary techniques used in GCF collection.

Reference	Analysis	Markers	Aim	Main findings
[12]	ELISA	IL-1*β*	To find the difference in the IL-1*β* levels between healthy control and CP patients. To find the relationship between clinical parameters and IL-1*β* levels.	The levels of IL-1*β* increased in accordance with progression of periodontal disease.

[13]	ELISA	Resistin	To measure and compare the levels of resistin in GCF in healthy subjects, chronic periodontitis, and diabetes mellitus type 2 (T2DM) patients.	The level of resistin increased in CP and T2DM patients. Hereafter, the level of resistin in GCF could be considered as a biomarker for periodontitis in T2DM patients.

[14]	ELISA	TNF-*α*	To measure TNF-*α* levels in GCF and in serum, and to find the effect of periodontal disease on TNF-*α* levels.	TNF-*α* level in GCF could be used as a biomarker for periodontal disease.

[15]	ELISA	Monocyte chemoattractant protein (MCP-1)	To determine MCP-1 levels in GCF, serum, and saliva, and to evaluate the effect of periodontal therapy on MCP-1 levels.	GCF and saliva MCP-1 levels could be used as biomarkers to indicate the severity of periodontal disease.

[16]	ELISA	Prostaglandin E2 (PGE2)	To evaluate PGE2 levels in GCF in healthy subjects and patients with periodontal disease, before and after treatment.	The levels of PGE2 positively correlated to the severity of periodontal disease.

[17]	SPM	Alkaline phosphatase (ALP)	To compare GCF ALP levels in patients with CP before and after nonsurgical periodontal treatment.	GCF ALP levels could monitor the periodontal disease status and effect of nonsurgical periodontal treatment.

[18]	SPM	ALP	To measure the GCF ALP levels in different periodontal disease stages.	ALP levels increased with periodontal disease progression. Thus it could be considered a good biomarker for periodontal disease progression.

[19]	ELISA	Oncostatin M (OSM)	To determine the level of OSM in GCF of gingivitis and CP patients and to evaluate the effect of periodontal treatment on level of OSM.	Levels of OSM correlated to the clinical periodontal parameters (PD and CAL) and could be used as a biomarker for periodontal disease.

[20]	ELISA	Cortisol	To measure the levels of salivary and GCF cortisol in anxious and nonanxious patients with CP.	Anxiety had a positive effect on periodontal disease and the levels of cortisol in GCF can be considered a biomarker for CP.

[21]	ELISA	Plasma glutathione peroxidase (eGPx)	To determine the eGPx levels in GCF to clarify the effect of oxidants and antioxidants on periodontal disease.	There was a positive correlation between the levels of eGPx in GCF and periodontal diseases. eGPx could be considered as a marker of oxidative stress in periodontal diseases.

[22]	ELISA	MCP-1	To evaluate the MCP-1 role during the development of periodontal disease and to evaluate the outcome of periodontal treatment on the levels of MCP-1.	The levels of MCP-1 elevated in accordance with the severity of periodontal disease. Periodontal treatment reduced the MCP-1 levels in GCF.

[23]	ELISA	MCP-1, IL-18	To examine MCP-1 and IL-18 GCF levels in control and periodontally diseased patients.	IL-18 and MCP-1 levels positively correlated to periodontal disease status.

[24]	ELISA	Pentraxin-3 (PTX3)	To evaluate PTX3 levels in Plasma and GCF in subjects with and without periodontal disease.	PTX3 levels in GCF could be considered as a biomarker for periodontal disease.

[25]	EIA	Neopterin	To determine if neopterin levels in GCF correlated with periodontal clinical parameters.	Neopterin increased proportionally with the severity of periodontal disease and it could be considered as a biomarker of periodontal disease.

[26]	ELISA	C-reactive protein (CRP)	To measure the levels of CRP in different periodontal disease stages.	Levels of GCF CRP and serum CRP elevated proportionately to periodontal disease severity.

[27]	EIA	Leukotriene B4 (LTB4)	To evaluate the relationship between periodontal clinical parameters and GCF LTB4 levels from diseased sites, previous to and after treatment of periodontitis.	GCF LTB4 levels increased with the severity of periodontal disease and reduced after nonsurgical periodontal treatment.

[28]	Sandwich enzyme immunoassay kit	Vascular endothelial growth factor (VEGF)	To find the correlation between GCF VEGF levels and the periodontal clinical parameters. To find if there was a correlation between the levels of VEGF in GCF and serum.	There was a positive correlation between the levels of VEGF in GCF and the periodontal clinical parameters. The same correlation was observed between the levels of VEGF in GCF and serum.

[29]	ELISA	Visfatin	To determine the concentrations of visfatin in GCF and serum in control and periodontally diseased patients in the presence and absence of T2DM.	Positive associations were observed between the levels of visfatin and periodontal disease in all study groups.

[30]	ELISA	IL-17, IL-18	To discover the role of IL-18, IL-17 in different periodontal disease stages before and after treatment.	IL-18 levels in GCF were found to correlate with periodontal disease severity, and periodontal treatments caused a decline in its concentration. IL-17 was not detected in the GCF.

[31]	ELISA	VEGF	To determine the level of VEGF in different periodontal disease stages and to explore the effect of treatment on VEGF levels in GCF.	Levels of VEGF in GCF elevated in relation to periodontal disease severity. Periodontal therapy led to a decrease in their levels.

[32]	ELISA	Visfatin	To measure the serum and GCF visfatin levels. To explain the role of scaling and root planning on visfatin levels.	Visfatin levels increased in accordance with disease progression and could be used as biomarkers during the treatment of periodontal disease.

[33]	Enzyme assay	ALP	To determine the existence and ALP levels activity in GCF in different stages of periodontal disease.	There was a relationship between periodontal disease and ALP level.

[34]	ELISA	Cystatin C	To measure the level of cystatin C in serum and GCF in different periodontal disease stages.	Cystatin C levels in serum and GCF correlated to the severity of periodontal disease and reduced after treatment.

[35]	ELISA	Osteopontin (OPN)	To measure the relation between clinical parameters and osteopontin (OPN) levels in GCF. To evaluate the effect of periodontal treatment on OPN levels.	GCF OPN levels increased with the severity of periodontal disease and the treatment resulted in a decrease in OPN levels.

**Table 3:** Summary of absorption techniques used in GCF sampling.

Reference	Analysis	Markers	Aim	Main findings
[36]	Enzyme assay (fluorimetric MMP kit)	MMP-1, -2, -3, -8, -9, -12, -13	To measure the levels of MMP in children with and without aggressive periodontitis (AgP).	The levels of MMP were raised in AgP sites compared to nondiseased sites in the same subjects.

[37]	ELISA	Myeloid related protein (MRP) 8/14, MRP14, total protein	To determine if the total protein, MRP14, and MRP8/14 in GCF can differentiate healthy from periodontitis sites in CP patients and if they could differentiate healthy subjects from CP patients.	These markers could not differentiate healthy from periodontitis sites in CP patients, but their levels in CP patients were higher than in healthy subjects.

[38]	Bradford method	Protein carbonyl (PC)	To assess GCF and serum levels of PC in patients with CP.	There was an increase in PC levels among CP patients, more than in healthy subjects.

[39]	ELISA, automatic colorimetric method	Total oxidant status (TOS), RANK ligand (RANKL), osteoprotegerin (OPG)	To explore the levels of total oxidant status (TOS), OPG, and RANKL levels in GCF and serum in different periodontal disease stages.	TOS, OPG, and RANKL levels increased with the severity of periodontal disease.

[40]	ELISA	Calprotectin, osteocalcin, cross-linked N-terminal telopeptide (NTx)	To evaluate the levels of osteocalcin, NTx, and calprotectin in GCF among healthy, G, CP, and generalized aggressive periodontitis (GAgP) patients.	Calprotectin level in GCF was considered as a marker for periodontal disease, while osteocalcin and NTx levels could indicate abnormal bone turnover.

[41]	ELISA	IL-1*β*, IL-6, IL-11, OSM, leukemia inhibitory factor (LIF)	To determine the concentrations of IL-1*β*, IL-11, IL-6, OSM, and LIF in GCF and plasma among periodontally diseased patients.	IL-1*β*, IL-11, and IL-6 GCF levels increased, but not plasma levels. They were considered dependable inflammatory biomarkers in periodontal diseases.

[42]	ELISA	Soluble triggering receptor expressed on myeloid cells 1 (sTREM-1)	To evaluate sTREM-1 levels in GCF of subjects with and without GAgP or CP and their association with subgingival plaque bacteria.	Elevated sTREM-1 levels at diseased sites and their positive association with clinical and microbiologic parameters strengthen the correlation of this marker with periodontitis.

[43]	ELISA, quantitative time-resolved immunofluorometric assay (IFMA)	MMP-8, MMP-13, tissue inhibitor of matrix metalloproteinase- (TIMP-) 1	To compare GCF levels of MMP-13 and -8 and TIMP-1 in periodontitis patients with and without RA.	RA did not affect the clinical periodontal parameters.

[44]	ELISA	RANKL, OPG	To determine the level of OPG and RANKL in GCF after nonsurgical periodontal treatment.	It could be a good indicator of treatment success.

[45]	ELISA	IL-33	To determine if IL-33 levels in GCF, saliva, and plasma could be used to differentiate between healthy and CP patients.	IL-33 levels could not be used as a biomarker for periodontal disease.

[46]	Electrochemiluminescence technique	Osteocalcin	To measure saliva, plasma, and GCF osteocalcin levels and correlate them with osteoporosis and periodontitis.	GCF osteocalcin levels were associated with periodontal disease only.

[47]	ELISA, multiplexed bead immunoassay (MPBI), SPM	IL-1*β*, -18, elastase, MMP-8, -9	To evaluate the effect of scaling and root planning on periodontal status and on the levels of IL-1*β*, elastase, MMP-9, and MMP-8 in patients with and without T2DM.	Scaling and root planning reduced the levels of IL-1*β*, elastase, MMP-9, and MMP-8 in both groups.

[48]	ELISA	TNF*α*, RANKL	To measure the TNF*α* and RANKL concentrations in GCF of patients with AgP, after photodynamic or nonsurgical periodontal treatment.	Both types of treatment had the same influence on TNF-*α* and RANKL concentrations.

[49]	MPBI	IL-6, -4, -10, -13, -17, TNF*α*, macrophage inflammatory protein- (MIP-) 1*α*, MIP-1*β*, MCP1, regulated on activation normal T-cell expressed and secreted (RANTES)	To determine the effect of adjunctive sub-antimicrobial-dose doxycycline (SDD) on the local inflammatory response through chemokine and cytokine levels in GCF samples from CP patients.	SDD aided nonsurgical periodontal therapy.

[50]	ELISA	MMP-8, TIMP-1	To determine the effect of azithromycin in addition to scaling and root planning in the treatment of periodontal disease.	Azithromycin did not present any advantage over scaling and root planning.

[51]	ELISA	Mucosa-associated epithelial chemokine (CCL28), IL-8, IL-1*β*, TNF-*α*	To determine the concentrations of CCL28, IL-1*β*, IL-8, and TNF-*α* in GCF among study groups.	CCL28, IL-1*β*, IL-8, and TNF-*α* concentrations were elevated in accordance with the severity of periodontal disease.

[52]	Flow cytometry	TNF-*α*, IL-1*β*, -6, -8, -10, -12p70	To estimate the outcome of periodontal treatment on GCF and serum concentrations of many cytokines related with periodontal disease and premature birth.	GCF cytokine level reduced significantly after periodontal treatment.

[53]	MPBI, ELISA	Pentraxin 3, IL-10, -1*β*, -6, -8, TNF*α*	To estimate the correlation between clinical periodontal measurements and the concentrations of six cytokines.	There was a strong correlation between periodontal status and PTX3 or IL-1*β* levels in GCF.

[54]	ELISA	MDA, SOD, melatonin	To determine GCF concentrations of superoxide dismutase (SOD), malondialdehyde (MDA), and melatonin in GAgP and CP patients as oxidative stress biomarkers.	SOD, melatonin, and MDA could be used to differentiate between GAgP and CP patients.

[55]	Fluorometric kits	MMP-1, MMP-2, MMP-3, MMP-8, MMP-9, MMP-12, MMP-13	To measure GCF MMPs levels after localized aggressive periodontitis (LAgP) treatment.	LAgP treatment with SRP and systemic antibiotics was active in reducing local levels of specific MMPs.

[56]	ELISA	MMP-8, MMP-9, MMP-13	To evaluate whether the presence of periodontitis and metabolic syndrome was related to MMP in GCF in the Korean community.	MMP (-13, -8, -9) individually correlated to the presence of periodontitis and metabolic syndrome.

[57]	Western immunoblot	MMP-13	To determine the role of GCF MMP-13 in adult CP patients.	There was significant increase in MMP-13 action in advanced periodontal disease.

[58]	ELISA	IL-23	To determine GCF IL-23 levels in healthy subjects and patients with periodontal disease.	IL-23 levels increased correspondingly to periodontal disease progression.

[59]	ELISA	TNF*α*, soluble TNF receptors 1 and 2	To evaluate TNF-*α* level, soluble TNF receptors 1 and 2 in serum and GCF of healthy and CP patients.	The levels between the two TNF receptors were disproportionate.

[60]	ELISA, immunoturbidimetric analysis	Stem cell factor (SCF), high-sensitivity C-reactive protein (hs-CRP)	To determine the relation between GCF and serum concentration of hs-CRP and SCF of two CP groups of which one is with T2DM and the other is without.	SCF and hs-CRP concentrations increased in patients with T2DM.

[61]	ELISA	Calprotectin	To evaluate the levels of calprotectin in GCF in GAgP patients prior to and after periodontal treatment.	Levels of calprotectin were indicators of disease activity in both subject and site levels.

[62]	ELISA	Myeloperoxidase (MPO), calprotectin	To observe calprotectin levels in GCF during therapy for GAgP. To determine a correlation between the MPO and calprotectin which were also determined.	Levels of calprotectin in GCF correlated to periodontal disease severity and decreased in concentration after treatment.

[63]	HPLCG	Platelet activating factor (PAF)	To determine the correlation between PAF and periodontal healing.	Alterations in PAF levels in GCF might be valuable for observing the regeneration and repair of periodontal tissues.

[64]	ELISA	chondroitin sulfate (CS), ALP	To determine the role of CS, ALP levels in estimating different periodontal disease stages.	The level of CS was better than the ALP level for determining periodontal disease stages.

[65]	ELISA	CS WF6 epitope	To evaluate GCF levels of CS WF6 epitope in healthy and periodontally diseased patients.	CS WF6 epitope levels positively correlated to the advancement of periodontal disease.

[66]	ELISA	MMP-8, -9, OPG, CRP, IL-1*β*	To evaluate the performance of MMP-8, -9, OPG, CRP, and IL-1*β* levels in GCF as a biomarker for periodontal disease.	MMP-8, -9, OPG, CRP, and IL-1*β* levels could support clinical parameters in the diagnosis of periodontal disease.

[67]	ELISA	IL-1*β*, IL-8, MMP-8	To evaluate the influence of SRP on levels of cytokines in GCF from CP patients, in relation to clinical parameters.	SRP reduced the IL-8, IL-1*β*, and MMP-8 GCF levels.

[68]	ELISA	MMP-8	To determine the association between MMP-8 in GCF and the severity of periodontal disease.	The level of active MMP-8 was higher in sites with deeper pocket depth.

[69]	ELISA, immunoturbidimetry	MCP-4, hs-CRP	To investigate GCF and serum levels of hs-CRP and MCP-4 among healthy and periodontally diseased patients.	hs-CRP and MCP-4 levels increased from periodontal healthy to periodontitis. hs-CRP and MCP-4 could be biomarkers of inflammation in periodontal health and disease.

[70]	ELISA	MCP-1, TNF-*α*	To determine and correlate GCF levels of MCP-1 and TNF-*α* in CP and AgP patients.	GCF levels of MCP-1 and TNF-*α* showed positive correlation.

[71]	ELISA, fluorometric method	PGE2, thiobarbituric acid reactive substance (TBARS)	To evaluate the effects of SRP and flurbiprofen in smokers and nonsmokers in CP patients on two GCF biomarkers.	PGE2 and TBARS levels in smokers decreased more than in nonsmokers after the flurbiprofen intake.

[72]	IFMA	MMP-8	To measure the levels of MMP-8 in GCF among two CP groups (smokers and nonsmokers).	The levels of MMP-8 could be used in the monitoring of periodontal diseases.

[73]	ELISA, IFMA	Azurocidin, chemokine ligand 5, MPO, TIMP-1 MMP-13, -14	To determine the diagnostic accuracy of GCF biomarkers. To compare two analytical techniques used to measure MMP-8 levels.	Collagenolytic MMPs and myeloperoxidase (MPO) could be considered as good biomarkers for periodontal diseases. IFMA analytical method was more precise than ELISA.

[74]	ELISA, radioimmunoassay	25-Hydroxy vitamin D3, osteocalcin, IL-1*β*, IL-6	To investigate the effect of SRP on the levels of 25-hydroxy vitamin D3 and three other biomarkers in GAgP patients.	Periodontal treatment led to reduction in the levels of 25-hydroxy vitamin D3 and IL-1*β*.

[75]	ELISA	PGE2, IL-1, TNF-*α*	To estimate the effect of combining two antibacterial drugs in initial periodontal treatment on periodontal parameters and certain biomarkers in patients with aggressive periodontitis.	Both types of treatment had substantial effect on periodontal disease status.

[76]	ELISA, immunoblotting	hCAP18/LL37, CS	To quantify GCF levels of hCAP18/LL-37 and CS in healthy, CP, and AgP study groups.	A positive correlation between the CS and hCAP18/LL-37 levels was noted in CP patients only.

[77]	MMP-8 specific chair-side dip-stick test	MMP-8	To determine the accuracy of MMP-8 specific analytical techniques.	This testing method could be useful to support clinical periodontal diagnosis.

[78]	ELISA, SPM	MMP-8, -9, TIMP-1, -2, MPO	To determine GCF levels of five biomarkers in healthy and CP patients before and after treatment.	The biomarker levels were greater in CP groups. Their levels reduced after treatment.

[79]	ELISA	LL-37	To evaluate GCF LL-37 levels in control and periodontally diseased groups and the degree of LL-37 by GCF elements.	LL-37 was detected in both study groups. There was high degradation of LL-37 level, mainly in *Porphyromonas gingivalis* positive sites.

[80]	MPBI	Granulocyte macrophage colony stimulating factor (GM-CSF), interferon-*γ* (INF-*γ*), IL-10, IL-1*β*, IL-2, IL-6, TNF-*α*	To determine the outcome of periodontal treatment by monitoring the alterations in cytokine levels from GCF samples in GAgP patients.	The periodontal treatment led to an increase in IL-10 levels and reduced IL-1*β* and GM-CSF levels.

[81]	ELISA	8-Hydroxydeoxyguanosine	To evaluate the effect of nonsurgical periodontal treatment on 8-hydroxydeoxyguanosine levels in GCF and saliva.	8-Hydroxydeoxyguanosine in GCF could reveal the severity of periodontal disease.

[82]	MPBI	INF-*γ*, IL-4, IL-33, thymic stromal lymphopoietin (TSLP)	To measure GCF levels of TSLP, IFN-*γ*, IL-4, and IL-33 in healthy and periodontally diseased patients.	Levels of IFN-*γ* related to the site stage and not on the disease stage IL-4. TSLP levels were detected in a few patients, while IL-33 was not detected.

[83]	SPM	ALP	To explain the effect of nonsurgical periodontal treatment on ALP action in GCF among CP patients.	ALP showed high activity following periodontal treatment, but after 60 days the ALP action reduced.

[84]	ELISA	IL-1*β*, TNF-*α*, MMP-8, MMP-9	To determine the effect of nonsurgical periodontal treatment together with photodynamic therapy (PDT) on periodontal conditions in CP patients.	The use of PDT did not show any benefit in nonsurgical periodontal treatment.

[85]	ELISA	Visfatin	To identify the existence of visfatin in serum and GCF.	The level of visfatin increased in relation to the severity of periodontal disease.

[86]	ELISA	8-Isoprostane	To measure 8-isoprostane concentrations in GCF in different periodontal diseases.	8-Isoprostane concentrations elevated in accordance with periodontal disease progression.

[87]	ELISA, RANDOX analyzer	Progranulin, hs-CRP	To measure GCF and serum levels of progranulin and hs-CRP in control subjects, CP and CP with T2DM patients.	CP with T2DM patients showed more hs-CRP and PGRN levels than the other groups.

[88]	ELISA	MMP-9, MMP-8	To measure GCF MMP-9 and MMP-8 levels in healthy subjects and patients with periodontal disease.	GCF MMP-9 and MMP-8 showed elevated levels in periodontally diseased patients.

[89]	ELISA	MMP-2, MMP-8	To measure GCF levels of MMP-9 and MMP-2, and the MMP-8 levels in saliva among control subjects and patients with periodontal diseases.	All the types of MMP were found to be associated with clinical parameters.

[90]	ELISA, Western blot radioimmunoassay	IL-1*β*, MMP-8, bone resorption marker carboxyterminal telopeptide cross-link fragment of type I collagen (ICTP), total collagenase activity	To discover the association between specific biomarkers in GCF with bone resorption clinical parameters.	The biomarkers were associated with clinical attachment loss.

[91]	MPBI	IL-1*α*, -1*β*, -6, -10, TNF-*α*	To measure the total GCF levels of six cytokines in patients with periodontal disease before and after nonsurgical periodontal therapy.	Nonsurgical periodontal treatment resulted in reduced IL-1*β*, -1*α* and IL-6 levels. Nonetheless, TNF-*α* or IL-10 levels were not decreased.

[92]	MPBI	IL-1*β*, IL-4, IL8, elastase activity	To observe differences in clinical, immunologic, and microbiologic responses to SRP in patients with different periodontal diseases.	SRP resulted in nonsignificant differences between severe forms of CP and GAgP.

[93]	ELISA	IL-1*β*, IL-8, MMP-8, MMP-9	To measure the concentration of specific biomarkers in GCF and the bacterial compositions in dental plaque in patients with and without type 1 diabetes (T1DM).	IL-1*β* and MMP-8 concentrations were found to be more elevated in patients with T1DM.

[94]	ELISA	IFN-*γ*, IL-23, IL-4, IL-17, TNF-*α*, OPG, RANKL	To determine the outcome of complete mouth SRP and noncomplete mouth SRP on cytokines levels and on clinical parameters over a twelve-month period.	Both types of treatment showed improvement in clinical parameters and the same changes in cytokines at twelve months.

[95]	ELISA	RANKL, OPG	To determine OPG and RANKL levels in GCF in patients with CP and AgP, as well as healthy subjects.	RANKL was present in periodontitis sites, especially in moderate periodontitis patients, whereas OPG was not noticeable in some sites with bleeding on probing.

[96]	IFMA, MMP-8 specific chair-side dip-stick test, DentoAnalyzer Device, ELISA	MMP-8	To compare 4 techniques used for MMP-8 analysis.	DentoAnalyzer Device, IFMA and chair-side dip-stick test had the same detection ability, while dip-stick test appeared to be better.

[97]	ELISA	OPG, sRANKL	To determine GCF levels of the soluble RANKL and OPG in smokers with periodontal disease.	Smoking suppressed OPG production and led to increased sRANKL∖OPG.

[98]	Checkerboard immunoblotting	IL-1*β*, IL-8, MMP-8	To investigate GCF levels of three cytokines and the microbial composition of the subgingival biofilm in control group and patients with periodontitis.	There were more cytokines and bacteria in the nondiseased sites in patients with periodontal diseases than there were in healthy individuals.

[99]	MPBI	GM-CSF, IL-2, -10, -13, -6, -1*β*, TNF-*α*, IFN-*γ*	To observe the relation between subgingival bacterial species and GCF cytokine concentrations in periodontal health and GAgP.	GAgP patients showed elevated ratio of IL-1*β*/IL-10 compared to the control group.

[100]	ELISA, Erels' colorimetric method	IL-1*β*, TOS, total antioxidant status (TAS)	To investigate the smoking outcome on the relationship between oxidation and IL-1 in periodontitis patients and response to nonsurgical periodontal therapy.	SRP impacted IL-1*β* concentrations in GCF, while no effect was detected on the TAS and TOS.

[101]	ELISA	hs-CRP	To measure the concentrations of hs-CRP in GCF and serum in periodontally diseased patients in the presence and absence of coronary artery disease (CAD).	Both periodontally diseased groups showed higher Hs-CRPHs-CRP concentrations than did the control group.

[102]	MPBI	IL-2, 12(p70), -3, -4, -5, -10, -13, -1*α*, -1*β*, -6, -12(p40), -8, -7, -15, IP-10, MCP-1, MIP-1*α*, RANTES, eotaxin, IFN-*γ*, GM-CSF, TNF-*α*	To investigate the existence of GCF biomarkers among smokers and nonsmokers with and without periodontal disease.	Periodontitis patients showed increased biomarker profiles. Smoking led to a reduction in many chemokines and cytokines.

[103]	ELISA	Cystatin C, IL-1*β*, TNF-*α*	To determine cystatin C levels, IL-1*β*, and TNF-*α* in the GCF and saliva of periodontally healthy children (PHC) and children with gingivitis.	GCF and saliva cystatin C levels were higher in PHC, but there was no correlation between cystatin C levels and TNF-*α* or IL-1*β* levels in GCF or saliva.

[104]	ELISA	TNF-*α*, IL-4, INF-*γ*, IL-23, IL-17, sRANKL, OPG	To determine GCF levels of six biomarkers in CP patients with and without T2DM.	CP patients with T2DM showed more biomarker levels than did nondiabetic patients.

**Table 4:** Summary of studies to compare IL-1*β* concentrations (pg/ml).

References	Sample size	Study design	Healthy	Gingivitis	CP	GAgP
			Mean ± SD or range			
[41]	20H, 20G, 20CP, 20GAgP	Cross-sectional	49.81 (13.27 to 144.64)	45.68 (8.49 to 122.09)	128.99 (27.70 to 393.02)	93.78 (11.40 to 247.55)
[12]	30H, 30CP	Cross-sectional	195.7700		409.2733	
[51]	21H, 21G, 21CP, 21GAgP	Cross-sectional	36.44 ± 8.86	52.10 ± 7.15	423 ± 35.24	110.23 ± 9.20
[53]	50CP	Cross-sectional	0.20 ± 0.31 (healthy site)		4.93 ± 5.27 (diseased site)	
[66]	18H, 32G, 28 mild CP, 22 moderate-sever CP	Longitudinal investigation	118 (92–998)	482 (15–908) progressing disease activity		
[67]	21H, 30CP	Intervention	15.5 ± 14.0		72.5 ± 37.0	
[80]	25H, 24GAgP	Intervention	7.0 ± 3.9			19.3 ± 10.0
[98]	20H, 20CP	Cross-sectional	45.6 ± 35.0		98.8 ± 42.4	
[99]	25H, 31AgP	Cross-sectional	18.9 ± 8.4			36.3 ± 17.8
[103]	10H, 25G	Cross-sectional	14.0000	17.8732		

H: healthy subjects, G: gingivitis, CP: chronic periodontitis, GAgP: generalized aggressive periodontitis.

**Table 5:** Summary of studies to compare MMP-8 concentrations (pg/ml).

References	Samples	Study design	Healthy	Gingivitis	CP	GAgP
			Mean ± SD			
[67]	21H, 30CP	Intervention	2.6 ± 2.6		18.6 ± 6.4	
[88]	10H, 10CP	Cross-sectional	4.13 ± 12.32		15.13 ± 12.46	
[98]	20H, 20CP	Cross-sectional	14.1 ± 15.1		34.7 ± 30.0	
[11]	43H, 56CP	Cross-sectional	234.80 ± 169.71		240.24 ± 146.83	

**Table 6:** Summary of studies to compare TNF-*α* concentrations (pg/ml).

References	Samples	Study design	Healthy	Gingivitis	CP	GAgP
			Mean ± SD or median (range)			
[14]	20H, 20G, 20CP	Cross-sectional	3.02	90.22	82.94	
[53]	50CP	Cross-sectional	0.17 ± 0.31 (healthy sites in CP patients)		0.33 ± 0.33 (diseased sites in CP patients)	
[70]	20H, 20AgP, 25CP	Cross-sectional	0.34 (0.25 to 0.48)		0.71 (0.55 to 3.58)	1.03 (0.17 to 3.02)
[99]	25H, 31AgP	Cross-sectional	1.9 ± 1.4			2.0 ± 1.9
[103]	10H, 25G	Cross-sectional	27.690	32.072		
[80]	25H, 24GAgP	Intervention	1.9 ± 1.4			1.9 ± 1.8
[59]	16H, 22CP	Cross-sectional	0.32 ± 0.25		0.11 ± 0.13	
[91]	52CP	Intervention	0.01 (0.00–0.13) (healthy sites in CP patients)		0.06 (0.01–0.52) (diseased sites in CP patients)	

**Table 7:** Comparison of the mean of IL-1*β* concentration between different studies using the same analytical techniques.

Study	[41]	[12]	[51]	[67]	[103]
ELISA kit	Standard ELISA kit (Bender Med Systems, Vienna, Austria)	Standard ELISA kit (Immunotech, France)	Standard ELISA kit (RayBiotech)	Standard ELISA kit (Quantikine R & D System)	Standard ELISA kit (Biosource, Ontario, CA)

GCF collection method	Paper strips	Microcapillary tubes	Paper strips	Paper strips	Paper strips

Number of GCF samples	2	20 *µ*l	4	1	4

Number of subjects	20H, 20G, 20CP, 20GAgP	30H, 30CP	21H, 21G, 21CP, 21GAgP	21H, 30CP	10H, 25G

Mean and SD for IL-1*β* concentration (pg/ml) and clinical parameters for CP patients	**128.99**	**409.2733 (98.0503)**	**423.65 (35.24)**	**72.5 (37.0)**	
	PD = 5.24 (0.66)	OHI-S = 5.1567 (1.4343)	PD = 3.45 (0.46)	PD = 6.4 (0.6)	
	CAL = 5.44 (0.63)	GI = 1.7793 (0.4253)	CAL = 3.62 (0.34)	CAL = 5.4 (0.9)	
	PI = 3.91 (0.48)	PDI = 4.0333 (1.1592)	PI = 2.16 (0.28)	PI (%) = 64.4 (18.8)	
	PBI = 2.61 (0.32)	PD = 4.7667 (1.6333)	GI = 2.18 (0.34)	GI = 2.3 (0.8)	
		BOP = 2.0333 (0.8899)	BOP = 81.24 ± 14.20		

Mean and SD for IL-1*β* concentration (pg/ml) and clinical parameters for G patients	**45.68**		**52.10 (7.15)**		**17.8732 (10.0523)**
	PD = 2.51 (0.37)		PD = 2.36 (0.23)		PD = 1.3029 (0.3142)
	CAL = 0.66 (0.57)		CAL = 2.32 (0.18)		CAL = 1.3029 (0.3142)
	PI = 3.71 (0.57)		PI = 1.68 (0.24)		PI = 0.5563 (0.5410)
	PBI = 2.16 (0.33)		GI = 1.62 ± 0.28		GI = 0.4832 (0.4959)
			BOP = 71.24 ± 12.40		GBI = 0.1635 (0.1904)

Mean and SD for IL-1*β* concentration (pg/ml) and clinical parameters for H subjects	**49.81**	**195.77 (80.0795)**	**36.44 (8.86)**	**15.5 (14.0)**	**14.0000 (9.9482)**
	PD = 1.5 (0.17)	OHI-S = 2.0533 (0.1925)	PD = 1.64 (0.42)	PD = 1.5 (0.9)	PD = 1.0728 (0.0689)
	CAL = 0.04 (0.06)	GI = 0.2333 (0.4302)	CAL = 1.74 (0.42)	CAL ≤ 1	CAL = 1.0728 (0.0689)
	PI = 1.41 (0.39)	PDI = 0.2333 (0.4302)	PI = 1.28 (0.12)	PI (%) = 45.8 (12.7)	PI = 0.1883 (0.2346)
	PBI = 0.36 (0.22)	PD = 0.0000 (0.0000)	GI = 1.25 (0.11)	GI = 0.7 (0.3)	GI = 0.0000 (0.0000)
		BOP = 0.0000 (0.0000)	BOP = 5.80 (3.50)		GBI = 0.0000 (0.0000)

Mean and SD for IL-1*β* concentration (pg/ml) and clinical parameters for GAgP patients	**93.78**		**110.23 (9.20)**		
	PD = 5.03 (1.09)		PD = 3.83 (0.54)		
	CAL = 5.39 (1.97)		CAL = 3.93 (0.27)		
	PI = 3.55 (1.09)		PI = 2.42 (0.35)		
	PBI = 2.15 (0.91)		GI = 2.31 (0.44)		
			BOP = 86.41 (10.20)		

CAL = clinical attachment loss. PBI = papilla bleeding index. OHI-S = simplified oral hygiene index. GI = gingival index. PDI = periodontal disease index. PD = probing depth. BOP = bleeding on probing. GBI = gingival bleeding index. PI = plaque index.

**Table 8:** Comparison of the mean of MMP-8 concentration between different studies using the same analytical techniques.

Study	[67]	[88]
ELISA kit	Standard ELISA kit	Standard ELISA kit
	(Quantikine R & D Systems)	(Quantikine R & D Systems)

GCF collection method	Paper strips	Paper strips

Number of subjects	21H, 30CP	10H, 10CP

Number of samples	1	1

Mean and SD for MMP-8 concentration (pg/ml) and clinical parameters for CP patients	**18.6 (6.4)**	**15.13 (12.46)**
	PD = 6.4 (0.6)	PD = 6.9 (1.9)
	CAL = 5.4 (0.9)	CAL = 5.5 (1.3)
	PI (%) = 64.4 (18.8)	BOP (%) = 57.9 (14.6)
	GI = 2.3 (0.8)	

Mean and SD for MMP-8 concentration (pg/ml) and clinical parameters for H subjects	**2.6 (2.6)**	**4.13 (12.32)**
	PD = 1.5 (0.9)	PD = 2.5 (1.9)
	CAL ≤ 1	CAL = 1.9 (0.5)
	PI (%) = 45.8 (12.7)	BOP (%) = 5.32 (3.71)
	GI = 0.7 (0.3)	
